# Supplementary material for: Investigating the influence of physiologically relevant hydrostatic pressure on CHO cell batch culture
Source: Sci Rep. 2021 Jan 8;11:162. doi: 10.1038/s41598-020-80576-8 (PMC7794228; doi:10.1038/s41598-020-80576-8)
Supplement: Supplementary file 1 — Supplementary Information 1. [file 41598_2020_80576_MOESM1_ESM.zip › S1/IgG/Feb17 60 90 standard/Feb13 90mmHg 2.pdf]

Data File C:\Users\P...mStation\1\Data\TK_CHO_IgG 2019-02-17 14-04-01\016-3-90mmHg Feb13.D
Sample Name: 90mmHg Feb13

===

Acq. Operator	: SYSTEM	Seq. Line :	16
Sample Operator	: SYSTEM		
Acq. Instrument	: HPLC-0039	Location :	3
Injection Date	: 2/17/2019 3:09:23 PM	Inj :	1
		Inj Volume :	100.000 µl

Method : C:\Users\Public\Documents\ChemStation\1\Data\TK_CHO_IgG 2019-02-17 14-04-01\CHO_IgG_LC.M (Sequence Method)
Last changed : 2/8/2019 3:17:23 PM by SYSTEM

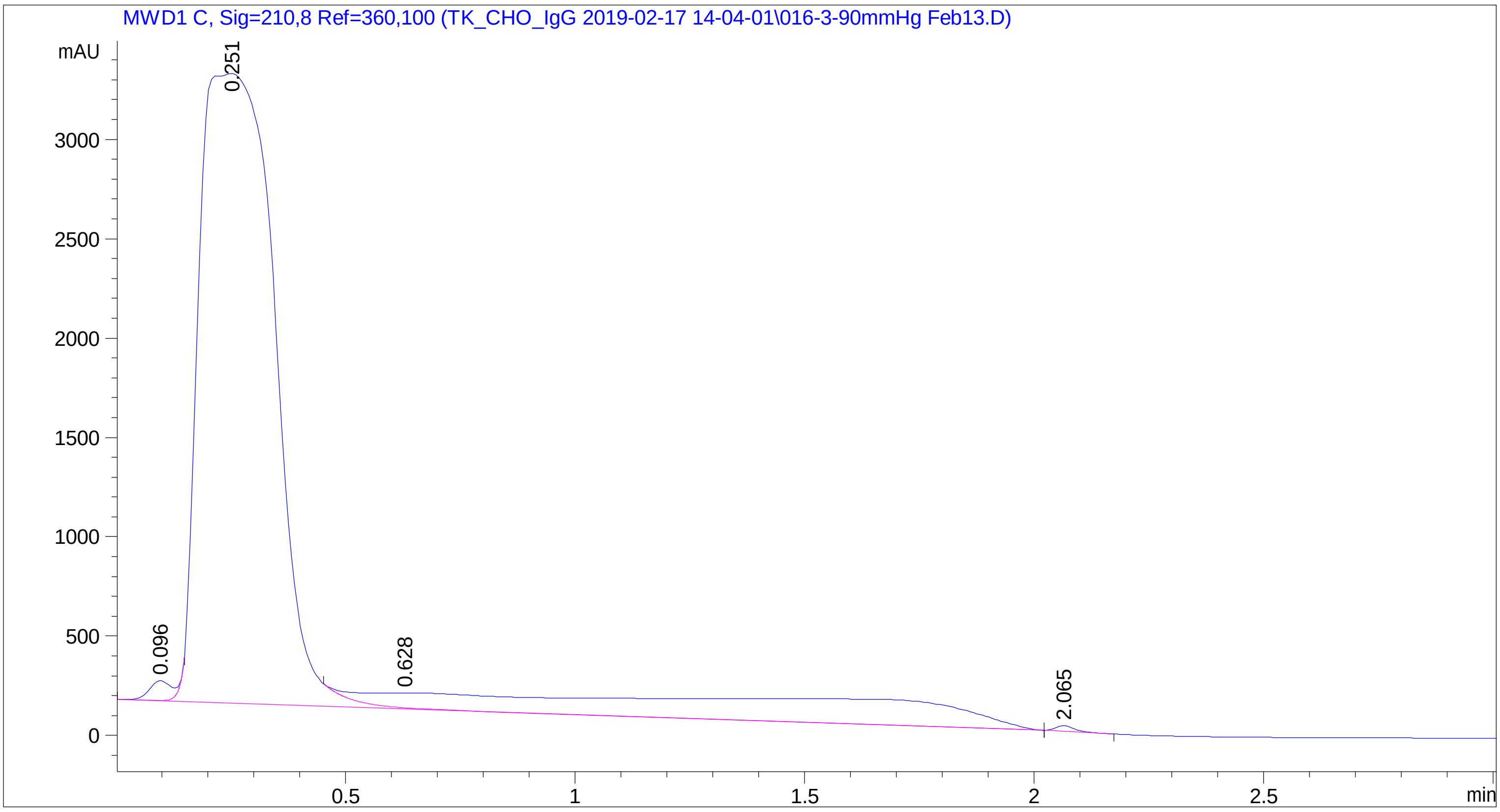

===

Area Percent Report

===

Sorted By : Signal
Multiplier : 1.0000
Dilution : 1.0000
Use Multiplier & Dilution Factor with ISTDs

Signal 1: MWD1 C, Sig=210,8 Ref=360,100

Peak #	RetTime [min]	Type	Width [min]	Area [mAU*s]	Height [mAU]	Area %
1	0.096	BV E	0.0503	331.10922	100.86928	0.7446
2	0.251	VV R	0.1661	3.58685e4	3167.19531	80.6586
3	0.628	VB E	1.3095	8184.14355	75.80694	18.4040
4	2.065	BBA	0.0479	85.74103	27.84342	0.1928

Totals : 4.44695e4 3371.71495

===

*** End of Report ***
